# Caffeic Acid Has Antiviral Activity against Ilhéus Virus In Vitro

**DOI:** 10.3390/v15020494

**Published:** 2023-02-10

**Authors:** Marielena Vogel Saivish, Carolina Colombelli Pacca, Vivaldo Gomes da Costa, Gabriela de Lima Menezes, Roosevelt Alves da Silva, Liliane Nebo, Gislaine Celestino Dutra da Silva, Bruno Henrique Gonçalves de Aguiar Milhim, Igor da Silva Teixeira, Tiago Henrique, Natalia Franco Bueno Mistrão, Victor Miranda Hernandes, Nathalia Zini, Ana Carolina de Carvalho, Marina Alves Fontoura, Paula Rahal, Lívia Sacchetto, Rafael Elias Marques, Maurício Lacerda Nogueira

**Affiliations:** 1 Laboratório de Pesquisas em Virologia, Departamento de Doenças Dermatológicas, Infecciosas e Parasitárias, Faculdade de Medicina de São José do Rio Preto, São José do Rio Preto 15090-000, SP, Brazil; 2 Brazilian Biosciences National Laboratory, Centro Nacional de Pesquisa em Energia e Materiais (CNPEM), Campinas 13083-100, SP, Brazil; 3 Laboratório de Estudos Genômicos, Departamento de Biologia, Instituto de Biociências, Letras e Ciências Exatas, Universidade Estadual Paulista, São José do Rio Preto 15054-000, SP, Brazil; 4 Faceres Medical School, São José do Rio Preto 15090-000, SP, Brazil; 5 Departamento de Biofísica e Farmacologia, Universidade Federal do Rio Grande do Norte, Natal 59072-970, RN, Brazil; 6 Unidade Especial de Ciências Exatas, Universidade Federal de Jataí, Jataí 75801-615, GO, Brazil; 7 Laboratório de Marcadores Moleculares e Bioinformática, Departamento de Biologia Molecular, Faculdade de Medicina de São José do Rio Preto, São José do Rio Preto 15090-000, SP, Brazil; 8 Department of Pathology, The University of Texas Medical Branch, Galveston, TX 77555, USA

**Keywords:** Ilhéus virus, caffeic acid, antiviral activity, inhibition, in silico analysis

## Abstract

Ilhéus virus (ILHV) is a neglected mosquito-borne flavivirus. ILHV infection may lead to Ilhéus fever, an emerging febrile disease like dengue fever with the potential to evolve into a severe neurological disease characterized by meningoencephalitis; no specific treatments are available for this disease. This study assessed the antiviral properties of caffeic acid, an abundant component of plant-based food products that is also compatible with the socioeconomic limitations associated with this neglected infectious disease. The in vitro activity of caffeic acid on ILHV replication was investigated in Vero and A549 cell lines using plaque assays, quantitative RT-PCR, and immunofluorescence assays. We observed that 500 µM caffeic acid was virucidal against ILHV. Molecular docking indicated that caffeic acid might interact with an allosteric binding site on the envelope protein.

## 1. Introduction

Ilhéus virus (ILHV) is a neurotropic mosquito-borne virus belonging to the genus *Flavivirus*, which includes other clinically relevant arboviruses such as the dengue (DENV), Zika (ZIKV), tick-borne encephalitis (TBEV), yellow fever (YFV), and Japanese encephalitis (JEV) viruses [1]. Ilhéus virus causes Ilhéus fever, an emerging zoonotic disease transmitted to humans predominantly through mosquito bites, especially *Psorophora* and *Ochlerotatus* genera [2]. Ilhéus virus was first isolated in 1944 from *Aedes* and *Psorophora* species collected in Ilhéus, Bahia state, in northeastern Brazil [3], and since then has been found circulating in the Amazon region (South and Central America), causing sporadic cases [4,5]. Humans, horses, and other mammals are probably dead-end hosts because of the short duration and low level of viremia they experience, ending the transmission cycle [6].

Ilhéus fever is an acute febrile disease similar to dengue fever, with typical clinical symptoms such as fever, headache, muscle and joint pain, skin rash, retro-orbital pain, nausea, vomiting, jaundice, sore throat, and abdominal pain [4,5,7,8,9,10,11,12,13]. In severe cases, the infection can progress to potentially fatal encephalitis and/or meningoencephalitis [7,12,13]. Laboratory diagnosis of ILHV infection may be difficult unless a virus isolate can be obtained because of cross-reactivity in serologic assays to other flaviviruses that circulate in the same area [14]. There is no information on how many individuals are infected with ILVH annually, but recently, a study revealed that between 1962 and 2021, there were more than 1000 cases in America, of which the majority came from the Amazon region in Brazil. It is estimated that up to 20% of symptomatic patients develop encephalitis, but this number is not exact due to the scarcity of detailed studies that mention this variable [15]. Furthermore, the number of cases is underreported and underestimated since public health systems do not conduct active searches to determine the circulation of this virus; cases are registered after an active investigation by university researchers. No vaccine or treatment is available for Ilhéus fever.

ILHV encodes three structural and seven nonstructural (NS) proteins [16] with molecular structures that are still not known. The structure, arrangement, and functions of ILHV proteins are assumed to resemble those of orthologues in viruses of the same genus, such as DENV or YFV. Flaviviruses enter host cells in a three-stage process that involves receptor binding, uptake by receptor-mediated endocytosis, and low pH mediated fusion between the viral and host cell endosomal membranes, releasing viral genomic RNA into the cell cytoplasm and initiating the viral replication cycle [17,18]. Much of this initial process is mediated by the virion envelope (E) protein, which is responsible for host cell receptor engagement as well as viral and host cell membrane fusion [18]. Recent advances in determining the structure of flaviviruses and their component proteins have focused on conformational and translational changes in the E protein as it transitions between the immature [19], mature [20], and fusion-active forms of the virus [21]. Specific surfaces on the E protein have been described as potential targets for structure-based antiviral drugs and alternate strategies for viral inhibition [22,23,24,25,26,27]. Although both monomer and dimeric E protein conformations can be recognized by mAbs, the dimer conformation is crucial for membrane fusion [28,29]. The essential nature of this entry step to the viral life cycle makes the E protein an obvious target for designing antiviral drugs.

Natural products present a rich source of molecules with diverse biological activities, with the potential to identify new drugs [30]. Caffeic acid (CA) is an organic compound found in almost all plants [31]. It is believed to exert a variety of pharmacological effects in the body, including antioxidant [32], antimicrobial [33], antiviral [29,34,35,36,37,38,39,40,41], and anti-inflammatory activity [42], but whether CA can interfere in flavivirus replication has not yet been explored. This study evaluates the antiviral activity of CA against ILHV.

## 2. Materials and Methods

### 2.1. Cells, Virus, and Chemicals

A549, HepG2, and Vero cells were cultured in Minimal Essential Medium (MEM) (Gibco, Waltham, MA, USA) supplemented with 10% (*v*/*v*) heat-inactivated fetal bovine serum (FBS) (Gibco, Waltham, MA, USA), 100 U/mL of penicillin, 0.1 mg/mL of streptomycin, and 0.5 µg/mL of amphotericin B (Gibco, Waltham, MA, USA), and were incubated at 37 °C in a humidified atmosphere containing 5% CO_2_. The C6/36 cells were cultured in Leibovitz-15 medium (L-15) with 10% FBS at 28 °C. Ilhéus virus (strain BeH 7445) stocks were propagated in C6/36 cells and titrated in Vero cells using the plaque-forming assay (PFU), described below in item 2.4. Caffeic acid (3,4-Dihydroxybenzeneacrylic acid) (Sigma-Aldrich, Saint Louis, MI, USA) was resuspended in dimethyl sulfoxide (DMSO) at 200 mM as a stock solution and stored at −20 °C until use.

### 2.2. Cytotoxicity Analysis

Briefly, 5 × 10^4^ A549, HepG2, or Vero cells grown in 96-well plates were treated with CA at concentrations ranging from 1000 µM to 31.25 µM for 48 h. Then, 1 mg/mL of 3-(4,5-Dimethyl-2-thiazolyl)-2,5-diphenyltetrazolium bromide (MTT) (Sigma, Aldrich, Saint Louis, MI, USA) was added to the cells, and they were incubated for one hour. Formazan crystals were dissolved in DMSO, and absorbance was determined at 550 nm using a Spectramax Plus Microplate reader (Molecular Devices, Sunnyvale, CA, USA). The results are presented as the percentage of viable cells compared to untreated control cells. All assays were independently performed three times in quadruplicate. From these results, the CC_50_ (cytotoxic concentration of the compound that reduced cell viability to 50%) was calculated from a dose–response curve in GraphPad Prism software (version 8.00) using four-parameter curve fitting.

### 2.3. Viral Infection Assays by Viral Titration

**Figure 1:**
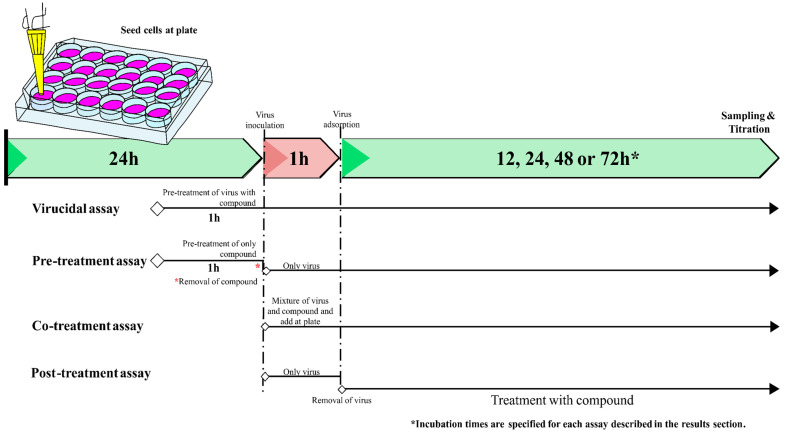
Scheme of the antiviral assay at different stages of virus infection.

Time-of-drug addition were performed to explore which steps of the ILHV replication cycle are blocked by caffeic acid. Briefly, CA was added to the virus and/or host cells at different times before, during, and after viral inoculation into the cells as follows (Figure 1): (1) pre-treatment of virus followed by inoculation of the treated virus into the cells investigates whether CA has virucidal or neutralizing activity; (2) pre-treatment of the cells with CA before viral inoculation explores whether this substance could block the viral receptor, inhibiting viral attachment to the host cells, or if it could induce production of antiviral host factors; (3) co-treatment of cells and virus during virus inoculation examines the function of CA during the steps of virus entry, including virucidal (neutralizing) activity and blockade of viral attachment and penetration into the cells; (4) treatment of virus-infected cells during the entire post-inoculation period investigates the antiviral effects of CA during post-entry steps such as genome translation and replication, virion assembly, and virion release from the cells. Viral infection experiments were performed in A549, HepG2, or Vero cells seeded in 24-well plates treated with CA or untreated controls. Under the different conditions described above, the cells were infected with MOI 1 of ILHV for one hour at 37 °C and revealed through the virus plaque-forming assay titration of supernatant or cell content (described in Section 2.4). Three independent experiments with quadruplicate measurements were performed. Data were analyzed by four-parameter curve fitting from a dose–response curve using GraphPad Prism (version 8.00) to calculate EC_50_ (concentration of the compound that inhibited 50% of the infection), and the selectivity index for the compound was calculated as the CC_50_:EC_50_ ratio.

### 2.4. Virus Plaque-Forming Unit Assay

Briefly, Vero cells grown in a 24-well culture plate were infected with 0.1 mL of ten-fold dilutions of supernatants. Following one hour of incubation at 37 °C, 0.5 mL of culture medium supplemented with 2% FBS and 1.5% carboxymethylcellulose sodium salt (Sigma-Aldrich, Saint-Quentin-Fallavier, France) were added, and the incubation was extended for three days at 37 °C. After removing the medium, the cells were fixed (formaldehyde 10%) and stained with 2% crystal violet diluted in 20% ethanol. Plaques were counted and expressed as plaque-forming units per milliliter (PFU·mL^−1^).

### 2.5. Plaque Reduction Assay

The plaque reduction assay was performed using a modified protocol previously described [43]. Briefly, A549 or Vero cells were seeded in 24-well plates at a density of approximately 1 × 105 and incubated at 37 °C in a humidified atmosphere containing 5% CO_2_ for 24 h. For the virucidal assay, a constant challenge of 50 PFU was treated with CA (1000–62.5 µM) in final volumes of 200 µL for 1 h at 37 °C. Then, these volumes were added to the confluent cell monolayers; plates were re-incubated for 1 h and gently shaken every 15 min for viral adsorption. Subsequently, the viral inoculum was removed, and a semi-solid overlay was added. For the co-treatment assay, a constant challenge of 50 PFU was treated with CA (1000–62.5 µM) in final volumes of 200 µL and immediately added to the confluent cell monolayers; plates were re-incubated for 1 h and gently shaken every 15 min for viral adsorption. Subsequently, the viral inoculum was removed, and a semi-solid overlay was added. For the post-treatment assay, 200 µL of the viral inoculum (50 PFU) were added to the confluent cell monolayers; plates were re-incubated for 1 h and gently shaken every 15 min for viral adsorption. Subsequently, the viral inoculum was removed, and a semi-solid overlay containing CA-decreasing concentrations (1000–62.5 µM) was added. For the virucidal, co- or post-treatment assays, control samples consisting of virus-infected untreated or treated cells were also included in each assay. Plates were maintained for three days in a humidified atmosphere containing 5% CO_2_ with daily observation. After this period, the overlay was removed, and cell monolayers were stained with a crystal violet solution, where non-stained lesions (plaques) were quantified and compared to the viral control. The percentage of viral inhibition was calculated by IP = [1 − (number of test plates/number of control plates)] × 100.

### 2.6. Immunofluorescence Assay

Vero or A549 cells were seeded onto 24-well plates (1 × 10^5^ per well) and incubated at 37 °C with 5% CO_2_ for 24 h. Cells were infected at MOI of 0.1 of ILHV, washed, and incubated with the compound for 24 h. Cells were washed and fixed in 4% paraformaldehyde, rewashed, and permeabilized with 0.05% Triton X-100 (JT Baker). Cells were blocked in 5% BSA (Sigma-Aldrich, USA) and incubated with an anti-flavivirus group antigen 4G2 antibody. AlexaFluor 488 Rabbit Anti-mouse IgG (Abcam, Waltham, MA, USA) and DAPI were used to stain viral proteins and cell nuclei, respectively.

### 2.7. RNA Extraction and RT-qPCR

Total RNA extraction was performed on cell culture samples using a Bio Gene Viral DNA/RNA Extraction kit (Bioclin, MG, Brazil) according to the manufacturer’s instructions. The RNA obtained from the samples was used as a template in a one-step real-time polymerase chain reaction (RT-qPCR) using primers (forward primer All_S 5′ TACAACATGATGGGGAARAGAGARAA 3′ and reverse primer All_AS2 5′ GTGTCCCAGCCNGCKGTGTCATCWGC 3′) targeting the NS5 gene of the *Flavivirus* genus, using GoTaq 1-Step RT-qPCR System (Promega, USA) [44]. Amplification was conducted in a QuantStudio 3 Real-Time PCR System (Thermo Fisher Scientific, Waltham, MA, USA) under the following conditions: reverse transcription at 45 °C for 15 min, denaturation at 95 °C for 10 min, and 40 cycles of 95 °C for 10 s, 60 °C for 30 s, and 72 °C for 30 s. A dissociation curve was then conducted, with a gradual increase in temperature from 60 °C up to 95 °C. The results were analyzed in QuantStudio 3 software v1.5.1 (Thermo Fisher Scientific, Waltham, MA, USA).

### 2.8. Data Analysis

Viral load at different time points with and without treatment was analyzed using ordinary two-way ANOVA with Sidak’s multiple comparison test; qPCR data were normalized into base 10 logarithm, and multiple t-tests with Holm–Sidak’s correction for multiple comparisons were applied. Viral loads in different treatment concentrations were compared using the Kruskall–Wallis test with Dunn’s multiple comparisons test. All values were expressed as mean ± SD from at least three independent experiments, and *p* < 0.05 was considered significant. All statistical data analyses were performed using GraphPad Prism software (version 6.0; GraphPad software, La Jolla, CA, USA).

### 2.9. E Protein Dimer Molecular Modeling and Molecular Dynamics

The Robetta server [45,46] was utilized for molecular modeling of the E protein as a dimer since this is the conformation that acts in membrane fusion, and it was expected to be more stable than the monomer. The genome sequence submitted to the server was retrieved from GenBank (accession number MH932545), and after modeling, the model protein was submitted to MolProbity [47] for quality analysis.

After validation of the model, the histidine pronation state for pH 7.4 was predicted using the PropKa server [48], and the initial steps for molecular dynamics (MD) were performed using GROMACS 5.1.2 [49] and the AMBER99SB-ILDN force field [50]. The MD protocol described here was previously executed in other studies [51,52,53]. The initial structure was inserted into a cubic box with a minimum distance of 10 angstroms (Å) between each box edge and the protein atom. This box was solvated with TIP3P water, and the system was neutralized with sodium ions.

The SETTLE algorithm [54] was used to maintain the internal rigid structure of the solvent molecules (water), while the solute covalent bonds involving hydrogen atoms were constrained by the LINCS algorithm [55]. The system temperature was set to 310 K (36.85 °C) and the pressure to 1 atm; both parameters were controlled by the V-rescale and Parrinello–Rahman algorithms [56,57], respectively. A cutoff of 1.0 nm was defined for nonbonded interactions, and the Ewald summation method for particle networks was used for long-range electrostatic interactions. The leapfrog algorithm was used to integrate the MD equations of motion with two fs as the time step.

Initially, the system was subjected to two energy minimization steps. The first was performed with 500 steps of the steepest descent algorithm and protein position restriction. The second minimization step utilized the same algorithm but with 10,000 steps and flexible water. After the minimization steps, the system was subjected to equilibration steps consisting of two 100 picosecond (ps) phases: an NVT ensemble and an NPT ensemble with protein position restriction for equilibration of the thermodynamic variables. The third and final equilibration phase was performed as an NPT ensemble of 1 nanosecond (ns) without protein restriction. Finally, the production run was performed as an NPT ensemble at 310 K with a total time of 100 ns. Trajectory analysis was performed using GROMACS 5.1.2. This work was performed in replicates (three replicates) to obtain a range of different conformations from independent MD simulations.

### 2.10. Molecular Docking and Interaction Analysis

Ensemble docking in this study was performed using all frames from each MD trajectory (replicate); each trajectory has 1000 frames. Docking analysis was limited to regions (including DI and DIII) reported as active site of the protein important for receptor binding and it has been described as an important target for viral-host fusion reaction [58,59,60]. Because we were working with dimeric E protein, two binding pockets (BP) to be docked by CA (molecular docking) were defined in each MD frame from three replicates (3000 frames) (See Figure S1 to observe both BP regions in one frame).

The protein and ligand were prepared for docking using the prepare_ligand.py and prepare_receptor.py scripts from MGLTools. The pH of the ligand was also adjusted to 7.4 using OpenBabel software.

The AutoDock Vina program (henceforth Vina) was used for molecular docking. One simulation was performed for each BP in each replicate, resulting in a total of 6000 runs of docking simulations. Each run generated nine ligand binding conformations, and the best conformational mode (best Vina score) was selected for further analysis.

All 6000 protein-ligand complexes for each run were submitted to BINANA software [61] for protein-ligand interaction analysis. The BINANA script was incorporated into a Python script we developed to analyze all complexes produced, and graphics were generated in RStudio. The BINAMA interaction parameters are presented in Table S1. A 2D interaction diagram was created using Discovery Studios Visualizer software (https://discover.3ds.com/discovery-studio-visualizer-download, accessed on 19 December 2022).

To determine the binding potential of CA, AutoDock Vina software was used to estimate binding free energy with experimental data from the Protein Data Bank, where CA is the inhibitor. This step was carried out by entering the argument ‘-score_only’ next to the receptor and ligand structure files on the Vina command line. The complexes were CA with ERK2 (PDB ID: 4N0S) and CA with HCAII (PDB ID: 6YRI).

## 3. Results

### 3.1. Caffeic Acid Is Not Toxic in Cell Culture

To evaluate the cytotoxic effect of caffeic acid, we set the maximum concentration for toxicity assessment to 1000 µM for the compound. The MTT cytotoxicity assay was used to determine A549, HepG2, and Vero cell toxicity at 72 h post-treatment [62]. Cell viability was well above 50% at the highest concentration (1000 µM), and as a result, we were unable to determine a CC50 value or selectivity index (SI) (Table 1).

### 3.2. Inhibition of ILHV Replication by Caffeic Acid Is Concentration-Dependent

**Figure 2:**
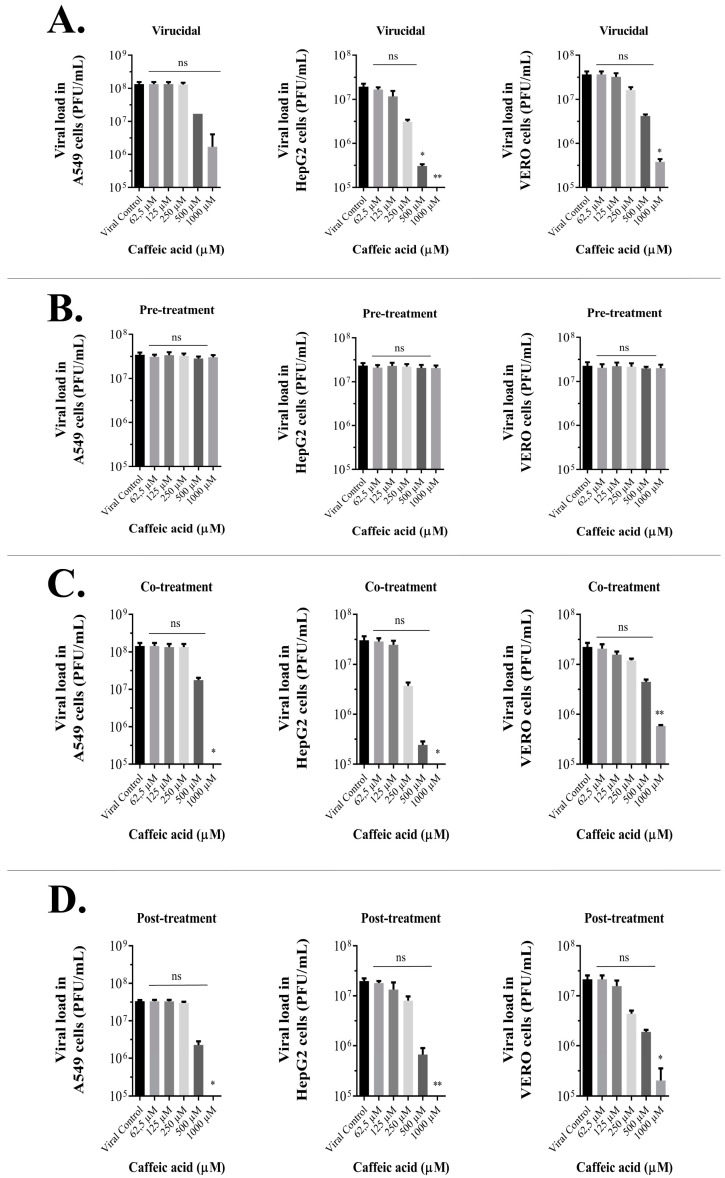
Caffeic acid’s effect on viral yield is concentration-dependent. ILHV production was measured in the presence of several dilutions of the tested compound in A549, HepG2, and Vero cells, with initial inoculum of MOI 1 under (**A**) virucidal, (**B**) pre-treatment, (**C**) co-treatment, or (**D**) post-treatment conditions 48 hpi. PFU infectivity titration of ILHV is shown in the left vertical axis. Error bars represent standard deviations. Values are the mean ± standard error obtained from three independent experiments. Asterisks indicate statistical significance between the control and each group as determined by Kruskall–Wallis test with Dunn’s multiple comparisons test. (* *p* < 0.05, ** *p* < 0.005).

To evaluate the impact of drug concentration on ILHV progeny reduction, we treated A549, HepG2, or Vero cells with different concentrations of CA under different treatment conditions and measured viral progeny production by viral titration 48 hpi (according to Section 2.3 and Section 2.4). To explore which steps of the viral cycle may be blocked by CA, time-of-drug-addition experiments were also performed. In brief, CA was added to the virus and/or host cells at different time points relative to viral inoculation of the cells (Figure 1). Viral progeny yields in non-treated cells reached 10^7^–10^8^ PFU/mL, depending on the treatment assay condition. However, a significant dose-dependent decrease in viral titers was seen in cells treated with CA (Figure 2A–D) except by no reduction was observed in the pre-treatment assay. For this reason, the pre-treatment condition was not further explored. The EC_50_ value is presented in Table 1. The presence of DMSO did not influence the production of progeny infectious virus particles under any assay condition (Figure S5).

Viral titer values were significantly reduced at concentrations of 1000 µM in three out of four conditions tested. In the virucidal assay, viral titers decreased with CA treatment at concentrations ranging from 500 to 1000 µM, and near-complete viral reductions were observed in the co-treatment and post-treatment assays with CA at 1000 µM. Such concentrations are high but were selected for the assay since CA did not show significant cytotoxicity at these concentrations, as observed in Table 1. These findings indicate that CA may have virucidal action and act in other processes related to viral entry or replication, thus resulting in lower viral titers observed in the co-and post-treatment assays, as observed in the virucidal assay.

### 3.3. Caffeic Acid Inhibited ILHV Lysis Plaque Formation in Cell Culture

**Figure 3:**
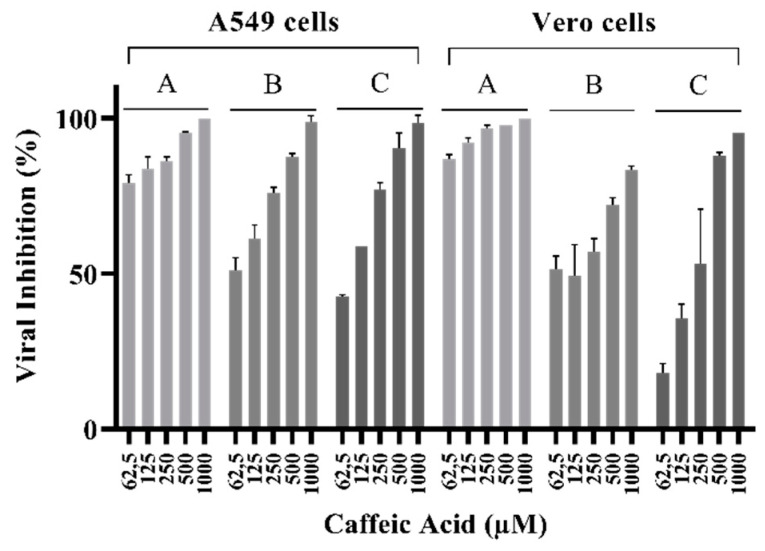
Plaque reduction potential of caffeic acid (62.5–1000 μM) on A549 or Vero cells infected with ILHV at 50 PFU/well. (**A**) Virucidal assay, incubating ILHV with caffeic acid at 37 °C for 1 h followed by addition to cell culture. (**B**) Co-treatment assay, simultaneously adding virus and caffeic acid to cell culture. (**C**) Post-treatment assay, infecting cells by incubation at 37 °C for 1 h followed by removal of virus and addition of caffeic acid post-infection. Viral control corresponds to 0% viral inhibition.

To explore the antiviral action of caffeic acid, we analyzed whether this molecule inhibited ILHV plaque formation by measuring the plaque-forming efficiency of the virus when different concentrations of CA were present. Plaque formation corresponds to a complete infection cycle, from entry to egress of newly formed viral particles. The inhibitory effect of the compound on ILHV expression was assessed in a dose-dependent manner (from 1000 to 31.25 µM of CA), with a constant challenge of 50 PFU per well, described in Section 2.5. After three days of ILHV infection, the A549 or Vero cells were fixed, and plaques were visualized by crystal violet staining and then quantified. Plaque formation was seen to be reduced for all concentrations of CA (Figure 3).

### 3.4. Caffeic Acid Is Virucidal against Ilhéus Virus

**Figure 4:**
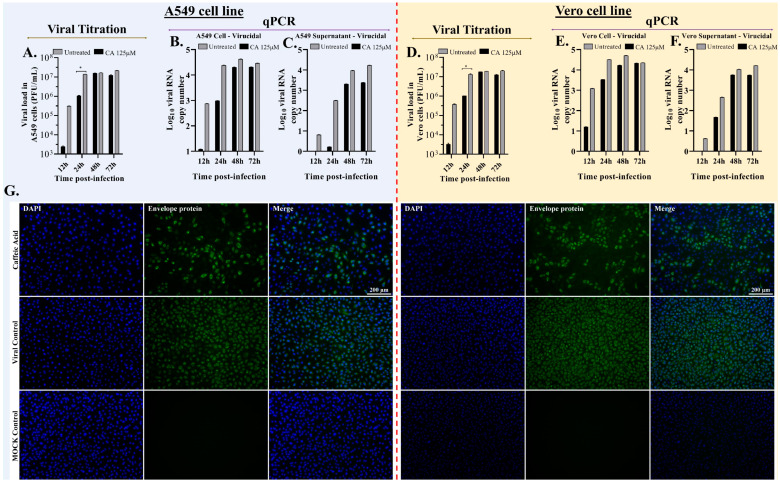
Virucidal assay of caffeic acid (CA). (**A**) A549 culture supernatants were harvested at the indicated times, and ILHV production was measured in the presence of 125 μM dilution of CA in cells infected with MOI 1 by viral titration and plaque-forming unit assay. (**B**) Total RNA viral quantification in cell content of A549 cells harvested at the indicated times; ILHV production was measured using real-time PCR in cell content in the presence of 125 μM dilution CA in cells infected with MOI 1. (**C**) A549 culture supernatants were harvested at the indicated times; ILHV production was measured in the presence of 125 μM dilution CA in cells infected with MOI 1 by real-time RT-PCR. Total RNA viral quantification in culture supernatants of A549 cells. (**D**) Vero culture supernatants were harvested at the indicated times; ILHV production was measured in the presence of 125 μM dilution of CA in cells infected with MOI 1, by viral titration and plaque-forming unit assay. (**E**) Total RNA viral quantification in cell contents of Vero cells harvested at the indicated times; ILHV production was measured in cell content in the presence of 125 μM dilution CA in cells infected with MOI 1 by real-time RT-PCR. (**F**) Vero culture supernatants were harvested at the indicated times; ILHV production was measured in the presence of 125 μM dilution CA in cells infected with MOI 0.1 by real-time RT-PCR. Total RNA viral quantification in culture supernatants of Vero cells. (**G**) ILHV was cultured in A549 or Vero cells with CA at 125 μM. The cells were challenged with MOI 0.1 of virus for 24 h. Mock (uninfected cells) and ILHV-infected cells without CA (viral control MOI 0.1) were used as controls. Expression of the ILHV envelope was detected using IFA, staining the 4G2 antibody with the 488 ALEXA antibody. (* *p* < 0.05).

To analyze whether CA directly affects ILHV infectivity, we infected A549, or Vero cells with ILHV (MOI 1) previously incubated for 1 h with 125 µM CA. The viral progeny was quantified by plaque-forming assay and qPCR in cell content and supernatant at the indicated times post-infection. In the assessment of viral RNA load in untreated infected cells and infected cells treated with CA, RNA viral copy numbers decreased by 1.79 log10 in treated A549 cell content (Figure 4B) and by 1.89 log10 in treated Vero cells (Figure 4D), with statistical significance (*p* < 0.05) at 12 h, and RNA viral copy numbers decreased during 24–72 h without statistical significance. At 12 h and 24 h, treatment with CA determined a decrease of 5.2/2.28 log10 RNA viral copy numbers in A549 cell supernatant (Figure 4C) and 0.48/0.98 log10 in Vero cell supernatant (Figure 4F), respectively, with statistical significance (*p* < 0.05), and RNA viral copy numbers decreased during 48–72 h without statistical significance. In the untreated infected cells with equivalent dilutions of DMSO without CA, viral titers and viral DNA loads were not inhibited by DMSO compared to the virus control (Figure S5).

To confirm the reduction of the virus titers, localizations of the E protein were monitored using an immunofluorescence assay 24 hpi as a qualitative assay. We detected viral E protein in the cytoplasm (green signal) of ILHV-infected cells without CA treatment under virucidal assay conditions using the anti-pan flavivirus E monoclonal antibody 4G2 stained with the 488 ALEXA secondary antibody; however, compared with the CA-untreated control, the viral E protein decreased in the cytoplasm of cells that received the virus previously incubated for 1 h with CA (Figure 4G). The infection performed in the immunofluorescence assay with a lower viral challenge (MOI 0.1) than that performed in the qPCR and viral titration assays (MOI 1) may have enabled a better visualization of the CA effect. As shown in Figure 2 and Figure 4A,D, CA affected viral infectivity, with the greatest decreases observed in viral titers until 24 h. This virucidal property increased according to CA dosage (Figure 2 and Figure 3), suggesting that CA impairs the infectivity of ILHV particles.

### 3.5. Caffeic Acid Reduces Ilhéus Virus Progeny Yield

**Figure 5:**
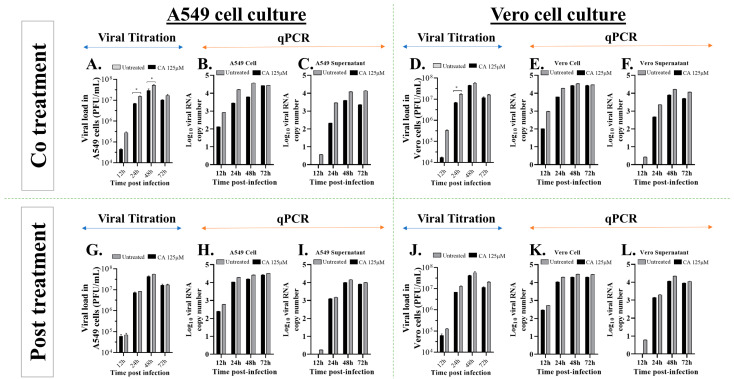
Antiviral activity of caffeic acid against ILHV investigated under different treatment conditions. (**A**,**D**,**G**,**J**) Culture supernatants were harvested at the indicated times; ILHV production was measured in the presence of 125 µM dilution CA in cells infected with MOI 1 by plaque-forming assay. (**B**,**C**,**E**,**F**,**H**,**I**,**K**,**L**) Culture supernatants and cell total RNA were harvested at the indicated time points. ILHV production was measured in the presence of 125 µM dilution of CA in cells infected with MOI 1, using real-time RT-PCR. (**A**) Viral load in co-treated A549 cells. (**B**) Viral RNA copy number in co-treated A549 cells content. (**C**) Viral RNA copy number in supernatant co-treated A549 cells. (**D**) Viral load in co-treated Vero cells. (**E**) Viral RNA copy number in co-treated Vero cells content. (**F**) Viral RNA copy number in supernatant co-treated Vero cells. (**G**) Viral load in post-treated A549 cells. (**H**) Viral RNA copy number in post-treated A549 cells content. (**I**) Viral RNA copy number in supernatant post-treated A549 cells. (**J**) Viral load in post-treated Vero cells. (**K**) Viral RNA copy number in post-treated Vero cells content. (**I**) Viral RNA copy number in supernatant post-treated Vero cells. Degree of significance indicated on the figure as follows: * *p* < 0.05.

To examine the effect of caffeic acid on ILHV replication, viral growth curves were conducted (at times 12, 24, 48, and 72 hpi.) in Vero or A549 cells in the presence or absence of CA treatment. The cells were co-treated or post-treated with 125 µM CA (Figure 5). Viral progeny production in the cell supernatants was quantified by plaque-forming assay at the indicated times post-infection (Figure 5A,D,G,J), and we observed a time-dependent increase in viral titers in non-treated cells. On the other hand, a slight reduction in viral yields was seen in the cells treated with CA, mainly in the co-treatment condition.

To confirm these results, we decided to investigate whether this molecule decreases ILHV RNA yield in co-treatment and post-treatment conditions. A549 and Vero cells were co-treated and post-treated with MOI 1, and cells and supernatants were collected at 12, 24, 48, and 72 hpi to measure viral progeny yields via qPCR (Figure 5B,C,E,F,H,I,K,L). These assays revealed that the application of CA promoted a discreet decline in ILHV progeny yields, but no statistically significant differences were observed.

**Figure 6:**
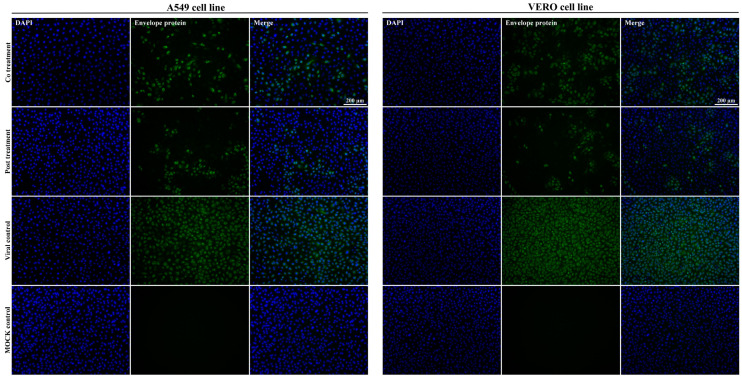
Immunofluorescence assay. Immunoflourescent images of ILHV-infected A549 or Vero cells under pre-treatment, co-treatment and post-treatment conditions with CA at 125 μM. The cells were challenged with MOI 0.1 of virus. Mock and ILHV-infected cells without CA were used as controls. Expression of the ILHV envelope protein was detected by IFA, staining 4G2 antibody with 488 ALEXA antibody.

We used immunofluorescence to assess the presence of viral antigens in infected cells with MOI 0.1 treated with CA and untreated infected cells. We found that treatment with CA led to a considerable decrease in cells with positive staining for the viral antigens (qualitative visualization of cells) (Figure 6). The infection performed in the immunofluorescence assay with a lower viral challenge (MOI 0.1) than that performed in the qPCR and viral titration assays (MOI 1 shown in Figure 5) may have enabled a better visualization of the CA effect. We ruled out the possibility that this effect on viral replication was the result of drug cytotoxicity since cell viability was assessed in Vero or A549 cells incubated with different concentrations of CA using the MTT method, and no significant decline in cell viability was seen after 72 h of incubation with the compound (Table 1). Together, these results indicate that CA affects Ilhéus virus progeny yields.

### 3.6. In Silico Analysis

The Robetta E protein dimer output model presented 97.7% of all residues in the favored region and 99.7% of all residues in the allowed regions (Figure S2). The MolProbity server indicated only three residue outliers, representing a high-quality protein model, especially considering a dimeric protein of 1002 amino acids. Additionally, the Clashscore (number of serious steric overlaps per 1000 atoms) showed a value of 0.67, representing the 99th percentile (where 100th is the best among structures of comparable resolution). When we superimposed the model with crystallographic E protein from DENV, WNV, ZIKV, and YFV, it can be noticed that the RMSD ranged from 0.866 Å to 1.021 Å (Table S2). These data suggest the model was well predicted based on experimentally solved structures.

**Figure 7:**
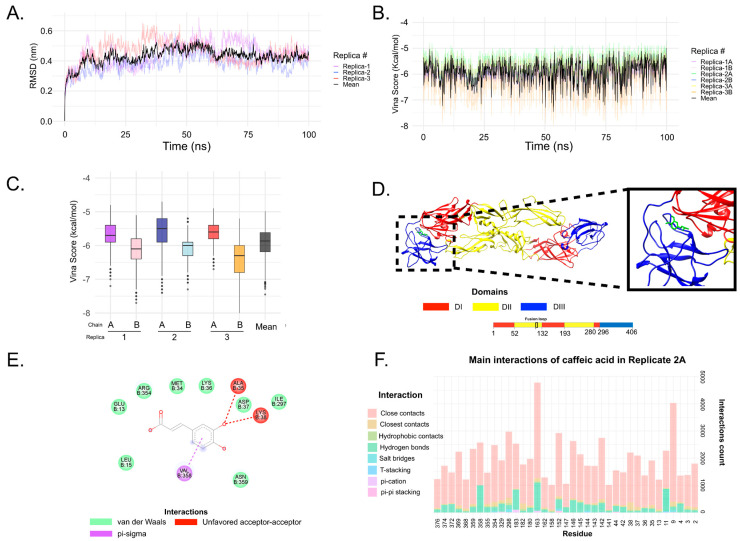
MD and ensemble docking analysis. (**A**) RMSD behavior throughout the 100 ns trajectory for all replicates. Replicates are shown as transparent colored lines, and the average for the five replicates is represented as a black line. (**B**) Vina score from caffeic acid docking in each frame for all trajectories. Details of values (mean, max, min, etc.) for RMSD and Vina score are presented in Tables S2 and S3. (**C**) Box plot of Vina Score distribution. Replicate 3B performed better (lower Vina Score values) than the other replicates/chains. (**D**) Caffeic acid (green line) binding between DI/DIII domains (red and blue ribbons, respectively). This is a complex retrieved from replica 2, chain A. (**E**) 2D diagram of interaction of protein-ligand complex from panel (**D**). This figure was created using Discovery Studios. (**F**) Main interaction residues of protein E, replicate 2A. Note that most of the residues are interacting in DI (residues 1–51; 132–192; 280–294) and DIII (residues 296–406). The interaction count below 1000 was not plotted. This analysis of the protein–ligand interaction was conducted using the BINANA algorithm and a Python script developed in-house. Analysis of the main interaction residues from all replicates and chains can be seen in Figure S4.

In the molecular dynamics simulations (Figure 7A), the pattern along the trajectory differed among replicates. Replicates 1 and 2 had similar behavior until 62 ns when RMSD increased in replicate 1. After ~62 ns, values for replicates 2 and 3 remained similar until ~85 ns. Replicates 1 and 3 presented similar RMSD values for the final 15 ns, with some stability; protein stability was not observed in RMSD for replicate 2 at the end of the trajectory. The cluster analysis using 0.3 nm as a cutoff is presented in Figure S3. This analysis shows a fluctuation in replicate 2 when there is an alternation between cluster 1 and cluster 4 after 75 ns of simulation. Similarly, this behavior after 75 ns is also observed in the cluster analysis for replicate 3. In general, cluster 1 (the most present throughout the simulation) appeared in all replicates near 50 ns. These results indicate very dynamic behavior by the E protein in solution, which can be expected since this protein has flexible hinges responsible for irreversible conformational changes during the viral cycle [63].

The mean average trajectory was 0.430 nm, extending up to 0.544 nm. Details of the RMSD values for each replicate can be found in Table S3. Differences between the replicates demonstrate the importance of working with independent MD simulations.

Although these differences were observed in the RMSD analysis, the AutoDock Vina score did not differ significantly between replicates (Figure 7B). Most lines fit the mean (Figure 7B, black line). The main differences occurred in replicates 2A and 3B. Vina score values ranged from −4.7 kcal/mol to −8.1 kcal/mol for all replicates. For each replicate, the lowest values ranged from −4.7 kcal/mol to −5.2 kcal/mol, and the highest values had wider ranges, from −6.7 kcal/mol to −8.1 kcal/mol. The average values (Figure 7B, black line) had the highest Vina score at −4.98 kcal/mol and the lowest at −7.45 kcal/mol.

When evaluating the energy distribution (Figure 7C) for all 1000 frames in each BP, chain B was seen to perform better (lower values) than chain A for all replicates, which underscores the importance of docking in both E protein chains rather than choosing just one. Energy profile details for each replica/chain can be seen in Table S4.

Figure 7D,E shows CA interacting between DI/DIII domains. However, not all dockings were focused on this region (Figure 7F). This is because the grid box was not restricted to the DI/DIII region but also included neighborhood residues. In the physiological environment, the ligand cannot be site-directed and is expected to bind to other protein regions. Docking analysis mainly evaluates whether the ligand has the potential to bind with great affinity in the region of interest. The main interactions of CA in all replicates/chains are depicted in Figure S4.

Among the results for the estimated free energy of binding in the experimental complexes containing CA, the predicted binding Vina scores for ILHV E protein were promising, with 75% (3rd quartile) of the scores lower than those observed for the experimental data for all replicates (Table 2). These results suggest that CA may be a promising inhibitor of the ILHV E protein.

## 4. Discussion

Emerging and re-emerging infectious diseases, especially virus-related diseases, have become an increasingly important concern in public health over recent decades. Accumulating knowledge in various areas against potential emerging viruses can help anticipate future epidemics, develop antivirals, raise alerts, and respond effectively to reduce adverse impacts on the general population. Members of the *Flaviviridae* family, such as ILHV, are RNA viruses with significant genetic plasticity and high mutation frequency [64]. They can acquire more virulent characteristics and greater transmissibility, expanding from poorly known etiological agents restricted to specific endemic regions to viruses with broad global distribution, as in the case of the Zika virus, with strains of greater concern [65,66]. Flavivirus infections are a major public health problem [67]; millions of people are infected yearly, and flaviviruses cause significant health, economic, and social burdens worldwide [6,68].

This study assessed the potential antiviral activity of CA against ILHV. Our results indicate that this natural compound can reduce replication in the tested virus in a dose-dependent manner. Previous studies suggest that CA has diverse biological functions including antibacterial [33], antiparasitic [69], anti-inflammatory [70,71], and antitumoral activity [72]. Another study also demonstrated that this molecule could inhibit HCV replication, which was mediated by the induction of HO-1 through the Keap1/Nrf2 pathway; this increased expression of HO-1 activated the IFNα antiviral response, thus blocking HCV replication [37]. Although this mechanism of action was not explored in the present study, a similar mechanism may be involved, and for this reason, we encourage further research along these lines.

Caffeic acid is a widely found phenolic compound from the hydroxycinnamate group derived biosynthetically from phenylalanine in plants. It occurs naturally in many agricultural products such as fruits, vegetables, wine, olive oil, and coffee [73]. Because processing plant foods yields by-products that are rich sources of phenolic compounds [74], and various natural compounds can directly affect the infectivity of viral particles, we explored the putative virucidal effects of CA on arboviruses. Caffeic acid was seen to strongly affect the infectivity of ILHV. These findings are supported by previous reports of other natural molecules, such as curcumin and luteolin, which demonstrated virucidal activity against ZIKV, CHIKV, and JEV, respectively [75,76].

Concentration-response antiviral treatment assays facilitated the testing of drug concentrations in multiple replicates, producing accurate EC_50_ values. In Vero cells, the EC_50_ values in the virucidal assay were higher than the EC_50_ values for the co-treatment and post-treatment assays. The results from the pre-treatment assay indicate that pre-incubation of the compound for a short period (2 h) followed by total removal of the drug may not be sufficient to generate a protective effect lasting long enough to influence later stages of the viral life cycle. Studies with a longer pre-incubation time for the compound may obtain different and perhaps more relevant results. Generally, EC_50_ values depend on the specific assay employed, and modifications (such as repeated treatment administration) could result in slightly different EC_50_ values. CA appears to have a greater viral inhibitory potential up to 24 post-infection. One possibility for decreased viral inhibition over time could be drug metabolization by cells. We encourage further studies to explore this possibility. Our viral growth curve assays in the presence or absence of 125µM CA demonstrated slight reductions in viral progeny and ILHV RNA. Perhaps these observed differences were discreet because the CA concentration used was below the EC_50_ values. Only a few cell lines permitted ILHV replication, including Vero and C6/36. A549 epithelial cells are permissive to infection by most flaviviruses [77,78,79,80] and offer a suitable model for studying host–virus interactions. In this research, we used A549 and HepG2 cells to confirm EC_50_ in Vero cells, making this the first study to demonstrate the activity of CA-based treatment against ILHV in human cell lines. The antiviral activity of CA was seen to differ slightly between cell lines; it has not yet been determined whether other cell lines are permissive to ILHV infection.

There is a dearth of studies that address plasma levels of CA in humans. A study was conducted to evaluate the bioavailability of caffeic acid status following red wine intake, where levels of up to 2.4 ng/mL were detected by HPLC analysis in plasma from people who ingested 300 mL of wine [81]. Another study, evaluating the bioavailability of caffeic acid status following coffee intake described plasma levels of ~80 nM [82]. Although these registered levels are below the values that we describe as being required to have an antiviral effect against ILHV, it is important to describe this CA activity since the bioavailability of drugs can be improved by formulating the drugs into nanosuspensions, solid lipid nanoparticles, polymeric nano- and microparticles, liposomes, niosomes, phytosomes, self-micro emulsifying drug delivery system, and microemulsion [83].

In addition to in vitro results, in silico data could help propose potential mechanisms of compounds that could act directly on viral proteins. Molecular dynamics simulations make it possible to study different E protein conformations. Most studies perform molecular docking using only one protein structure, which may not offer the most representative conformation; here, we used 3000 protein frames to evaluate the binding affinity of CA. This substance showed promising inhibition capacity by binding in regions previously studied for potential inhibitive action [58,59,84]. Finally, the AutoDock Vina results were better than crystallographic structures with CA acting as an inhibitor. Even so, our in silico findings indicate that CA offers strong potential against the envelope protein, which could explain the virucidal activity demonstrated by this substance. Previous studies proposed that compounds binding in this docking region (DI/DIII) addressed in our work—from the E protein of ZIKV, however—could lead to the interruption of the folding DIII back into the fusion loop and ultimately inhibiting ZIKV entry into host cells. In our work, as previously described in these studies, many important interactions were observed in the DIII region (see Figure 7F, residues between 296 to 406).

Natural molecules can disrupt viral replication at different stages of the viral life cycle, including viral entry, viral protein expression, assembly, and viral release [70,71,72]. We consequently performed a time experiment to determine the impact of CA on the virus life cycle in different treatment assays quantified by plaque assay. Our findings suggest that this compound could affect the early phase of virus replication and has a virucidal effect directly on the virus. This led us to investigate alternative mechanisms by which CA could prevent ILHV replication, and we assessed vRNA expression quantified by qPCR. These analyses suggest that CA could interfere with and decrease the expression of viral RNA in both cell lines and conditions tested. A similar effect was identified in HSV-1 in an in vitro study [85] when CA inhibited virus multiplication. An analysis of how the time of CA addition impacted its effects revealed that addition in the early post-infection period significantly inhibited the formation of infectious virus progeny in the infected cells, but after 6 hpi (i.e., the time needed for complete replication of the viral genome) did not effectively inhibit this process. Although our findings suggest that this compound may affect the immediate early or other phases of virus replication, we encourage further studies to be carried out, with different assays from those performed, so that a clear understanding of the action of CA is obtained. Due to several reports in the literature of cases of encephalitis in humans, it can be assumed that this virus is neurotropic. Therefore, studies involving neural cells may have a different outcome than that described here. We encourage further studies with different cell lineages for an in-depth understanding of metabolic pathways and mechanisms of action involved in these described antiviral activities.

## 5. Conclusions

Caffeic acid has demonstrated antiviral activities against ILHV in vitro. The antiviral effects were cell-type dependent, with a slight difference between A549, HepG2, and Vero cells. Cell line variability can result from differences in CA uptake or metabolic processing by the different individual cell lines. This work is the first antiviral study investigating ILHV. Our data open the door for further studies with CA using animal models for flavivirus infection.

## Figures and Tables

**Table 1:** Inhibition efficacy (EC_50_) and cell toxicity of caffeic acid in A549, HepG2, and Vero cell lineages.

	Inhibition Efficacy (µM) *			Cell Viability (%) *			
	EC_50_				A549	HepG2	Vero
	A549	HepG2	Vero	**1000** **µM**	72.4 ± 2.4	91.4 ± 5.2	100.5 ± 14.6
Virucidal	390.3 ± 32.4	140.6 ± 23.4	234.0 ± 39.2	**500 µM**	77 ± 6.6	96.2 ± 5.1	101.4 ± 9.7
Pre-treatment	Not achieved	Not achieved	Not achieved	**250 µM**	83.3 ± 6.8	94.7± 3.2	96 ± 9.2
Co-treatment	370.4 ± 19.9	167.9 ± 26.3	228.9 ± 58.4	**125 µM**	86.4 ± 4.3	96.5 ± 2.1	95.5 ± 7.6
Post-treatment	340.1 ± 34.4	187.4 ± 42.1	168.7 ± 33.3	**62.5 µM**	87.4 ± 11,2	93.8 ± 4.7	94.5 ± 7.2

* Values with 95% confidence intervals (CI).

**Table 2:** Binding energies from AutoDock Vina.

Vina Score from Ensemble Docking			
Structure	Vina Score (Kcal/mol)		
Replicate (Chain)	Min.	Max.	3rd Quartile
1 (A)	−7.20	−4.80	−5.40
1 (B)	−8.10	−5.10	−5.80
2 (A)	−7.40	−4.70	−5.20
2 (B)	−7.30	−5.20	−5.90
3 (A)	−6.70	−4.90	−5.40
3 (B)	−8.00	−5.20	−6.00
Average	−7.45	−4.98	−5.62
Estimated Binding Free Energy by Vina			
PDB ID	Vina Score (Kcal/mol)		
4N0S	−5.102		
6YRI	−4.273		

## Data Availability

Not applicable.

## Supplementary Materials

The following supporting information can be downloaded at: https://www.mdpi.com/article/10.3390/v15020494/s1. Figure S1—Representation of dimeric E protein, highlighting the two chains (chains A and B shown in blue and red, respectively). Figure S2—Ramachandran plot of E protein Robetta output model. Figure S3—Cluster analysis of MD simulation using a 0.3 nm cutoff. Figure S4—Main interaction E protein residues per replicate/chain. Figure S5—Activity of 0.5% DMSO under the ILHV. Table S1—BINANA interaction parameters. Table S2—RMSD comparison between E protein from ILHV and others Flavivirus with E protein structure experimentally solved. Table S3—Description of RMSD values. Table S4—Description of Vina docking values.
