# Supplementary material for: Caffeic Acid Has Antiviral Activity against Ilhéus Virus In Vitro
Source: Viruses. 2023 Feb 10;15(2):494. doi: 10.3390/v15020494 (PMC9961518; doi:10.3390/v15020494)
Supplement: Supplementary file 1 [file viruses-15-00494-s001.zip › viruses-2179978-supplementary.pdf]

Supplementary Material

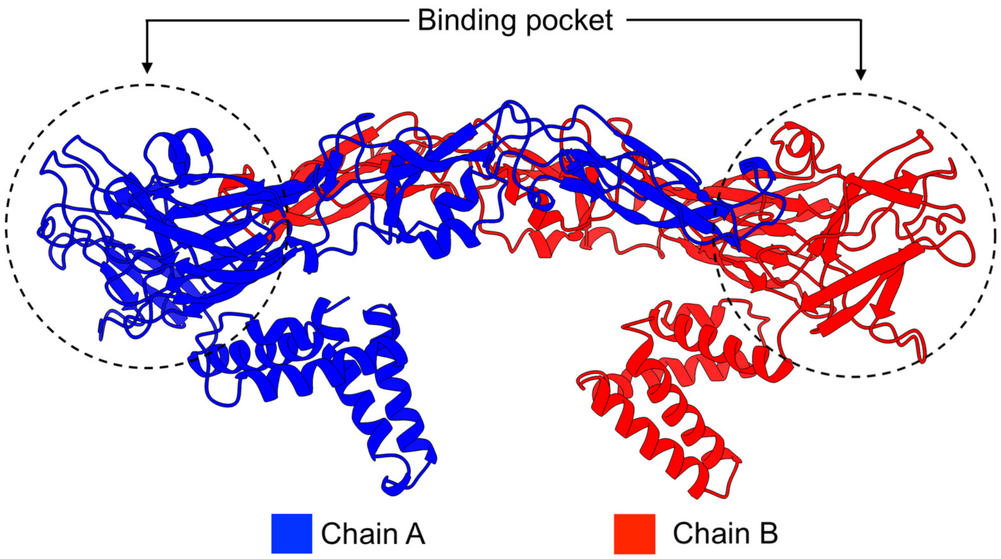

Sup. Fig. 1 – Representation of dimeric E protein, highlighting the two chains (chains A and B shown in blue and red, respectively). Each chain contains DI, DII, and DIII domains. AutoDocking Vina analysis was done in both chains (focusing on DI/DIII), as depicted in the circles of dashed lines in black.

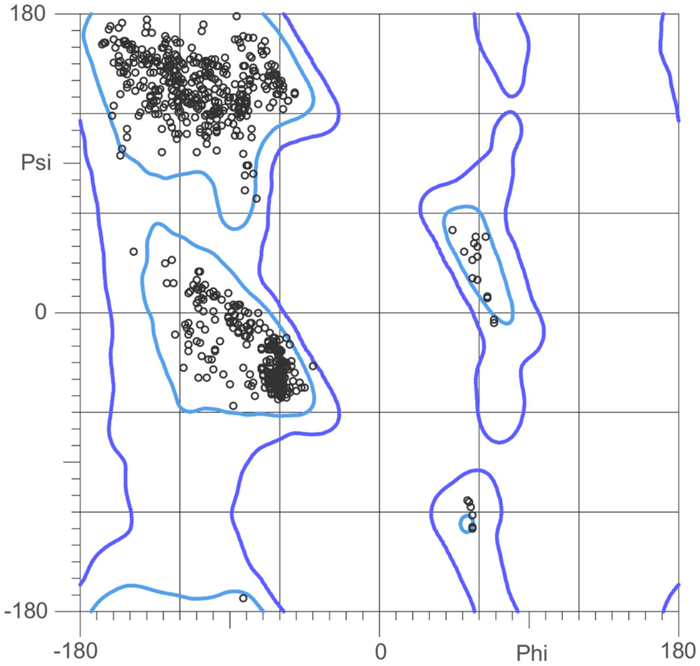

Sup. Fig. 2 – Ramachandran plot of E protein Robetta output model. The plot shows 97.7% of all residues in favored region (light blue line) and 99.7% of all residues in allowed regions (dark blue line).

Sup. Table 1 – BINANA interaction parameters

Parameters used to identify close contacts					
Closest contacts cutoff (Å)				Close contacts cutoff (Å)	
**2.5**				**4**	
Parameters used to identify hydrogen and halogen bonds					
Hydrogen bond dist (Å)		Hydrogen and halogen bond angle cutoff (Å)		Halogen bond dist cutoff (Å)	
**4**		**40**		**5.5**	
Parameters used to identify interactions between aromatic rings					
π Padding dist (Å)	π- π Interaction dist cutoff (Å)	π-Stacking angle tolerance (Å)	T-Stacking angle tolerance (Å)	T-Stacking closest dist cutoff (Å)	Catio- π dist cutoff (Å)
**0.75**	**7.5**	**30**	**30**	**5**	**6**
Parameters used to identify other notable interactions					
Salt bridge (Å)		Hydrophobic dist cutoff (Å)		Metal coordination dist cutoff (Å)	
**5.5**		**4**		**3.5**	

Parameters available at: https://durrantlab.pitt.edu/binana/

Sup. Table 2 – RMSD comparison between E protein from ILHV and others Flavivirus with E protein structure experimentally solved

**Virus**	**PDB ID**	**RMSD (Å)**
DENV	5N0A	1.021
ZIKV	5JHM	0.965
YFV	61W2	1.018
WNV	3IYW	0.866

Sup. Table 3 – Description of RMSD values

	RMSD (nm)						
Replicate	Min.	1st quartile	Median	Mean	3rd quartile	Max.	Sd[1]
1	0.0005	0.3933	0.4526	0.4483	0.5043	0.6877	0.0782518
2	0.000501	0.353320	0.388845	0.389054	0.424302	0.584991	0.0607297
3	0.000503	0.405428	0.454539	0.454159	0.507387	0.649971	0.0747719
Average	0.000501	0.408559	0.432122	0.430520	0.463375	0.544058	0.0524167

[1]Sd = standard deviation

Sup. Table 4 – Description of Vina docking values

Vina score from ensemble docking							
Structure	Vina score (Kcal/mol)						
Replicate (chain)	Min.	1st quartile	Median	Mean	3rd quartile	Max.	Sd[1]
1 (A)	-7.20	-5.90	-5.70	-5.71	-5.40	-4.80	0.364521
1 (B)	-8.10	-6.40	-6.10	-6.16	-5.80	-5.10	0.446784
2 (A)	-7.40	-5.90	-5.50	-5.61	-5.20	-4.70	0.511542
2 (B)	-7.30	-6.30	-6.00	-6.07	-5.90	-5.20	0.334031
3 (A)	-6.70	-5.80	-5.60	-5.63	-5.40	-4.90	0.299141
3 (B)	-8.00	-6.80	-6.30	-6.40	-6.00	-5.20	0.56715
Average	-7.45	-6.18	-5.87	-5.93	-5.62	-4.98	0.418215

Estimated binding free energy by Vina	
PDB ID	Vina score (Kcal/mol)
4N0S	-5.102
6YRI	-4.273

[1]Sd = standard deviation

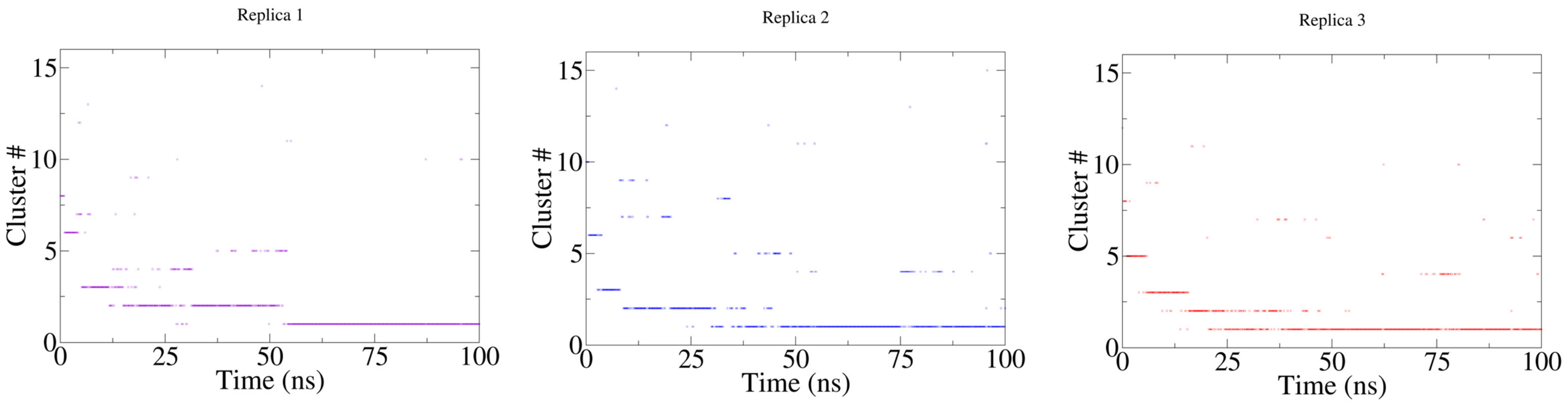

Sup. Fig. 3 – Cluster analysis of MD simulation using a 0.3 nm cutoff. The GROMOS algorithm was used for clusterization; this algorithm counts the number of neighbors using a cutoff, taking the structure with largest number of neighbors together with all its neighbors as a cluster and eliminating it from the pool of clusters. The process is repeated for the remaining structures in the pool. A total of 14, 15, and 12 clusters were found for replicas 1, 2, and 3, respectively.

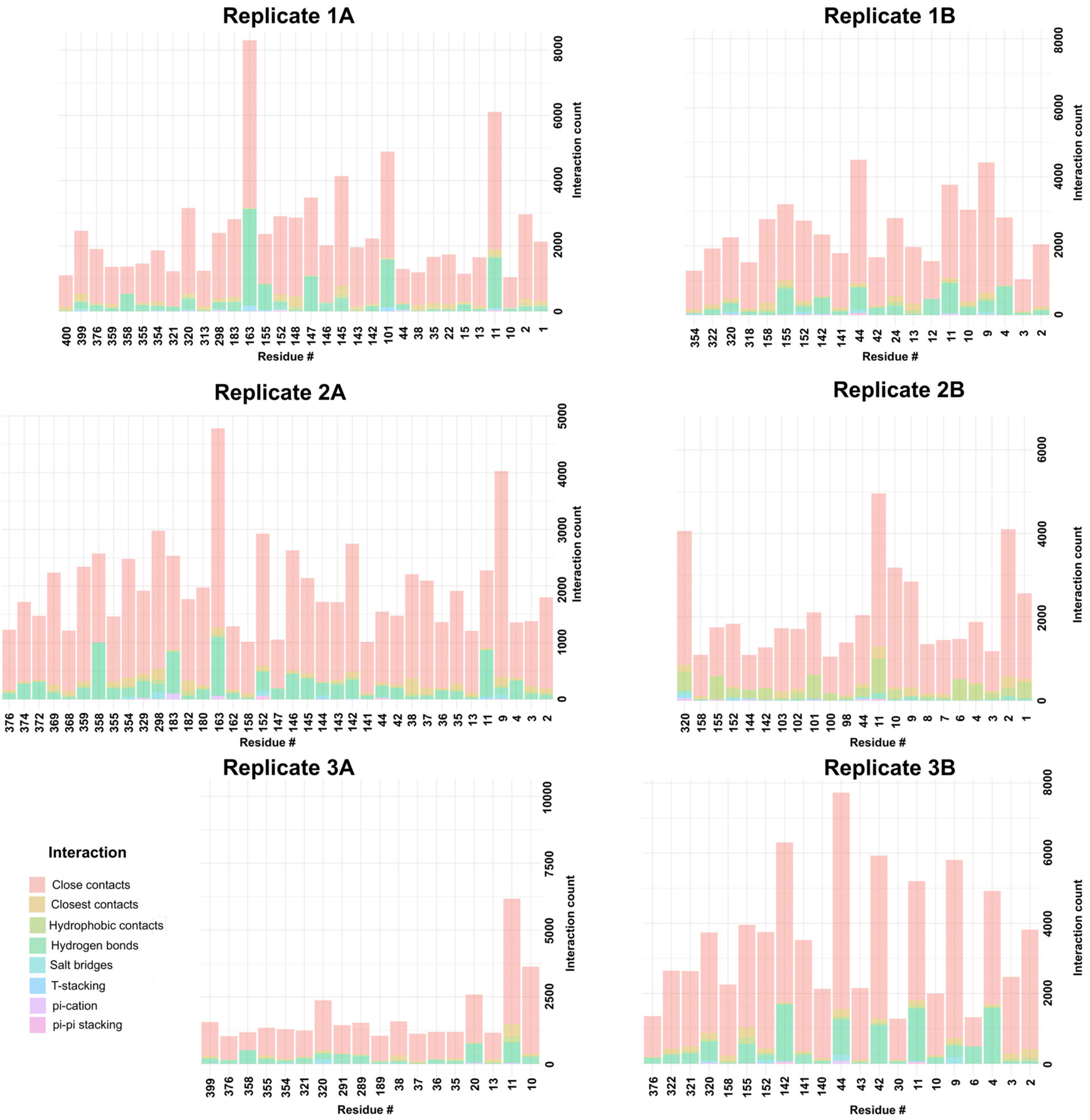

Sup. Fig. 4 – Main interaction E protein residues per replicate/chain. Residues with fewer than 1000 interactions were not depicted.

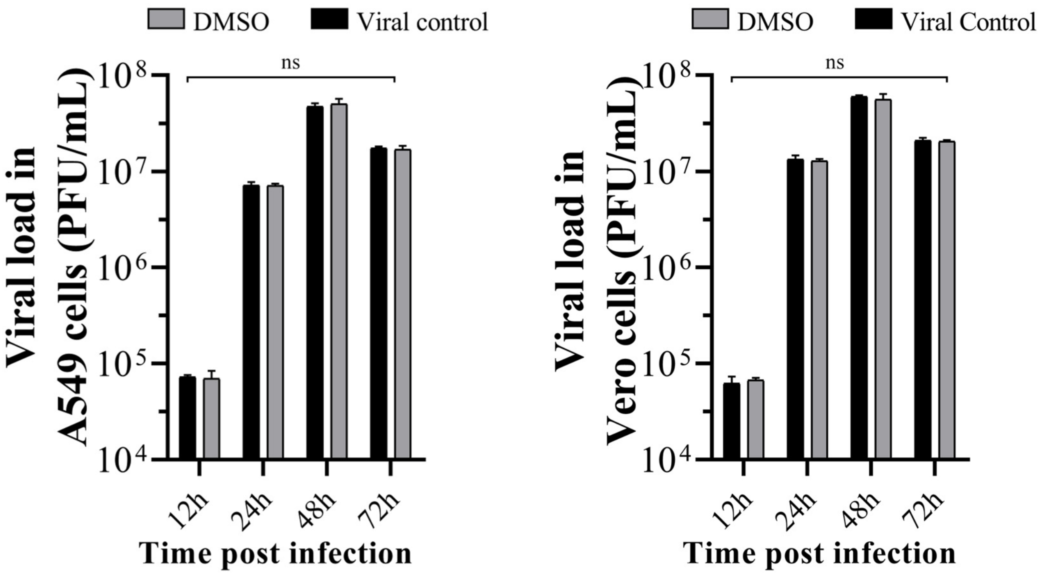

Sup. Fig. 5 – Activity of 0.5% DMSO under the ILHV. We evaluated the possible interference of DMSO on the viral progeny, since DMSO is the CA diluent used in our tests. We evaluated the action of 0.5% DMSO (this being the highest value used in our study, corresponding to the concentration of DMSO present in the 1000uM dilution of CA), comparing it with a viral control containing only MEM 2% FBS. There was no difference in viral progeny, shown in the graphs.
